# Real-time determination of sarcomere length of a single cardiomyocyte during contraction

**DOI:** 10.1152/ajpcell.00032.2012

**Published:** 2012-12-19

**Authors:** Pearu Peterson, Mari Kalda, Marko Vendelin

**Affiliations:** Laboratory of Systems Biology, Institute of Cybernetics, Tallinn University of Technology, Tallinn, Estonia

**Keywords:** heart muscle, sarcomere length, microscopy image analysis, autocorrelation analysis, fundamental period

## Abstract

Sarcomere length of a cardiomyocyte is an important control parameter for physiology studies on a single cell level; for instance, its accurate determination in real time is essential for performing single cardiomyocyte contraction experiments. The aim of this work is to develop an efficient and accurate method for estimating a mean sarcomere length of a contracting cardiomyocyte using microscopy images as an input. The novelty in developed method lies in *1*) using unbiased measure of similarities to eliminate systematic errors from conventional autocorrelation function (ACF)-based methods when applied to region of interest of an image, *2*) using a semianalytical, seminumerical approach for evaluating the similarity measure to take into account spatial dependence of neighboring image pixels, and *3*) using a detrend algorithm to extract the sarcomere striation pattern content from the microscopy images. The developed sarcomere length estimation procedure has superior computational efficiency and estimation accuracy compared with the conventional ACF and spectral analysis-based methods using fast Fourier transform. As shown by analyzing synthetic images with the known periodicity, the estimates obtained by the developed method are more accurate at the subpixel level than ones obtained using ACF analysis. When applied in practice on rat cardiomyocytes, our method was found to be robust to the choice of the region of interest that may *1*) include projections of carbon fibers and nucleus, *2*) have uneven background, and *3*) be slightly disoriented with respect to average direction of sarcomere striation pattern. The developed method is implemented in open-source software.

as an experimental model, isolated cardiomyocytes provide unique opportunities to study electrophysiology, mechanics, and bioenergetics. To bring this experimental model closer to the in vivo environment, mechanical contraction of cardiomyocytes can be induced through electrical stimulation and controlled by attached carbon fibers (23, 22). To induce a mechanical loading protocol on a single cardiomyocyte, carbon fibers can be moved leading to isometric or shortening contractions. However, similar to isometric contraction of the muscle fiber (10), it has been demonstrated that there is a shortening of sarcomeres in the middle of cardiomyocyte while the distance between carbon fibers or cell length stays constant (16, 5). Due to several technical difficulties in performing isosarcometric contraction experiments using real-time feedback, adaptive feed-forward control systems are commonly used to control the distance between carbon fibers leading to an inability to perform isosarcometric contraction experiments (16, 5). One of the technical problems in achieving real-time feedback control for isosarcometric experiments is the determination of the mean sarcomere length, the problem that is addressed in this work. While several methods exist, many of them suffer from inaccuracies. As demonstrated in this work and earlier by others (see below), methods based on autocorrelation function (ACF) and spectral analysis of microscopy images have several drawbacks that preclude their use in the real-time estimation of sarcomere length for feedback control of the contraction where the accuracy of control input such as the sarcomere length is of importance.

Whatever experimental technique is used for capturing data with sarcomere length information, various signal analysis methods have to be applied to estimate the mean sarcomere length of a cardiomyocyte under a microscope. A common task for all methods is to quantify some measure of repetition contained in captured data and relate this measure to the sarcomere length. For simplicity, we assume that the captured data are in the form of microscopy images acquired with a high-speed camera. This is also a practical simplification as transmission images with sarcomere length information additionally contain carbon fiber positional information that is needed for mechanical loading protocols. This information would not be readily available when using, for instance, laser-light diffraction-based techniques (7, 9, 11).

There are several requirements for the method of sarcomere length determination to make it useful in real-time control of cardiomyocyte contraction. First, it must be accurate at subpixel resolution to allow the usage of faster cameras that have smaller resolution parameters. Second, the method must be robust to a selection of region of interest (ROI) size. Using smaller ROI facilitates localized estimation of the mean sarcomere length as well as consumes less computational resources that may be essential for real-time control protocols. Because of the exact alignment of ROI to the sarcomere striation pattern is practically impossible due to continuous variations of the sarcomere orientations during contraction, the method must also be robust to the selection of ROI orientation. Third, the method must be robust in regions of ROI where the sarcomere signal is weak or even absent due to nonfavorable optical conditions and cell morphology or due to the presence of some external objects such as carbon fibers holding a cardiomyocyte. Finally, the method must have efficient implementation to ensure that the mean sarcomere length is determined before the next image frame from a high-speed camera arrives.

The sarcomeres appear in a transmission image of a cardiomyocyte as repeating patterns of darker and lighter regions of Z-disks and I-A-bands, respectively, forming a sarcomere striation pattern. The mean sarcomere length is defined as the spatial period of this pattern.

Several systems exist that can measure the mean sarcomere length from microscopy images in real time. The software SarcLen provided by IonOptix employs a fast Fourier transform (FFT) to determine the mean frequency of sarcomere spacing (Sarclen Algorithm, Technical Report, IonOptix, 2010). The software HVSL by Aurora Scientifica implements similar FFT-based algorithm as well as two ACF-based algorithms (Instruction Manual: Model 901A, Aurora Scientific, 2008). In the ACF-based algorithms, the sarcomere spatial frequency is determined either by applying an FFT-based algorithm to the ACF or by fitting it with a sine function with the frequency corresponding to the mean sarcomere spatial frequency. Both systems can measure sarcomere length at subpixel resolution by using quadratic approximation at the peak of the discrete frequency spectrum. However, in the FFT-based methods, the accuracy of the mean sarcomere length estimate strongly depends on a mismatch of pixel values at ROI ends due to the extension of ROI into periodic function, as FFT-based methods inherently do. In addition, the mismatch of pixel values at ROI boundaries changes during the cardiomyocyte contraction making it impossible to compensate such boundary effects, for example, by varying the signal length. These boundary effects can be observed, for example, in the mean sarcomere length evolution during the contraction: the sarcomere length rate may contain spurious peaks (Instruction Manual: Model 901A, Aurora Scientific, 2008), especially when measuring sarcomere length from a relatively small ROI. To suppress the boundary effects of the FFT-based methods, one can select a longer ROI that contains more sarcomeres and use Hann window filtering (4) or use ACF-based methods, as the above-mentioned commercial systems do.

The aim of this work is to develop an accurate and efficient computational method for determining the mean sarcomere length from transmission images of a single contracting cardiomyocyte and provide its implementation in an open-source software package. The developed method is first evaluated on using artificial signals with know periodicity and then is applied to a sequence of microscopy images of a cardiomyocyte taken during the contraction. Various sensitivity properties of the mean sarcomere length are analyzed to estimate the applicability of the developed method in practice. Finally, two example experiments are provided where the mean sarcomere length is estimated while varying preload and stimulation conditions.

## GENERAL DESCRIPTION OF THE MEAN SARCOMERE LENGTH DETECTION ALGORITHM

There are different kinds of errors that determine the accuracy of the mean sarcomere length estimated from a microscopy image: *1*) imperfect objective field caused by uneven illumination and presence of objects other than sarcomeres, *2*) restrictions and uncertainties in acquisition process leading to sampling errors and noisy data, and *3*) systematic errors from used algorithms.

This work aims at establishing the best algorithm that is robust to errors in input data and is exact for perfectly periodic input data, that is, the best algorithm must have no systematic errors.

### Main Algorithm

In simplified notation, as a basic algorithm to determine the mean sarcomere length of a cardiomyocyte, we propose to use least squares difference between the image and its shifted copy. Indeed, in the ideal case with the periodic signal on the image, shifting the image by one period would lead no difference between the original and shifted copy. In reality, we expect that the minima of the least squares difference will be obtained when the shift between image and its copy is close to the mean sarcomere length value. In the following text, we will denote the proposed algorithm as a “method of current work.”

For comparison of the results of current work we use two other conventional methods for estimating the mean sarcomere length.

The first method, labeled as the “Fourier spectrum” method, is based on calculating the power spectrum of a signal. The maximum point of the power spectrum is related to the fundamental spatial frequency of the signal, which, in turn, is inversely proportional to the fundamental period, i.e., the mean sarcomere length when applied on the transmission image of a cardiomyocyte. The second method, labeled as the “ACF” method, is based on calculating the ACF. The first positive maximum point of the ACF is related to the fundamental period of the signal and can be used to estimate the mean sarcomere length.

The main difference between the algorithm proposed in this work, and “Fourier spectrum” and “ACF” methods, is in the treatment of the boundaries of the image. Let us assume that the sarcomeres are aligned along the image lines. As it will be demonstrated in results, “Fourier spectrum” and “ACF” methods suffer from the difference of the signal on the opposing boundaries of the image leading to the systematic error in the mean sarcomere length estimation.

### Subpixel Resolution

In addition to the performance of the underlying algorithm, the accuracy of the mean sarcomere length is strongly influenced *1*) by the size of pixels of microscopy images and *2*) by the number of pixels containing sarcomere striation pattern data. These parameters are especially important because microscopy imaging provides a rather sparse representation of the sarcomere striation pattern. For example, in a typical microscopy image of a rat cardiomyocyte (see materials and methods), a single sarcomere unit is represented only by four or five pixel values per image line as determined be the ratio of a typical sarcomere length (1.8 μm) and pixel size (0.4 μm). On the other hand, the area within a microscopy image of cardiomyocyte where sarcomere striation pattern is more-or-less uniform and well visible, is always restricted, first, by the overall size of a cell; second, by variable morphology of the cell; and finally, by the presence of external objects such as carbon fibers used to fix the cell. Under these restriction of data acquisition, we aim at resolving the mean sarcomere length of a cardiomyocyte with subpixel resolution. For that we use piecewise linear representation of the object field under microscopy; compare this to the actual piecewise constant representation of the object field where the neighboring pixels are considered unrelated. By using linear interpolation we could derive analytical equations that allow us to estimate the mean sarcomere length with subpixel resolution using all three methods considered in this work.

### Image Filtering by Detrending

Assuming that the cardiomyocyte is aligned along the image lines, a typical line profile of a microscope transmission image of a cardiomyocyte contains variations that have different origins: sarcomere striation patterns, nuclei, external objects, etc. For our analysis, it is important to separate variations in *1*) image intensity induced by periodic intracellular structures and *2*) background. To decompose a sample sequence into the corresponding oscillatory and slowly varying parts, we use a simple but efficient method that takes advantage of knowing the spatial period of oscillations, that is, the initial estimate of the mean sarcomere length. For that, images are blurred with the kernel that has a half-width of ∼1/10 of sarcomere length, and through processing of local maxima, the oscillatory part is extracted. The performance of the algorithm is described in results and details are given in appendix.

## MATERIALS AND METHODS

This study develops a method for estimating the mean sarcomere length of a cardiomyocyte from a transmission image in real time. Here, experimental and numerical methods are described. A mathematically rigors description of the algorithms is given in appendix.

### Ethics Statement

Animal procedures were approved by the Estonian National Committee for Ethics in Animal Experimentation (Estonian Ministry of Agriculture).

### Cardiomyocyte Isolation

Adult outbred Wistar rats of both sexes weighing between 300 and 500 g were used in the experiments. Cardiomyocytes are isolated as described by Sepp et al. (20) with modifications from Jepihhina et al. (8).

### Solutions

Cells were imaged in a HEPES-Tyrode solution containing the following (in mM): 137 NaCl (71379; Sigma-Aldrich), 5.4 KCl (P5405; Sigma-Aldrich), 2.0 CaCl_2_ (21097; Sigma-Aldrich), 0.5 MgCl_2_ (63068; Sigma-Aldrich), 0.33 NaH_2_PO_4_ (Fluka Analytical; 71633), 5 HEPES (H3375; Sigma-Aldrich), and 5 glucose (158968; Sigma-Aldrich), pH 7.4 adjusted by NaOH (30531; Fluka Analytical) at 25°C.

### Imaging

Microscope experiments were performed on an inverted Nikon Eclipse Ti-U microscope (Nikon, Amstelveen, The Netherlands) equipped with a high-speed CCD camera (IPXVGA210-LMCN; ImperX), with a ×40 long working distance objective (CFI S Plan Fluor ELWD 40×/0.6; Nikon).

To ensure the immobility of cardiomyocytes during contraction we used a carbon fiber technique (22) with bidirectional control setup (5). Carbon fibers (Tsukuba Material Information Laboratory) that were mounted in glass capillaries (TW150–3; WPI, Sarasota, FA) were attached to an isolated cardiomyocyte using two micromanipulators (PatchStar Micromanipulator; Scientifica, East Sussex, UK). For manipulating carbon fibers, nanopositioners (Nano-OP100; MCL) were mounted to micromanipulators. For field stimulation, we used stimulator (module 2100; A-M Systems). For example experiments, the carbon fibers were coated with a biological adhesive, MyoTak (IonOptix, Dublin, Ireland), to improve their attachment to cardiomyocytes.

Solution with cells was placed on a cover glass of 0.15 mm in thickness (CS-24/50; Warner Instruments, Harvard Apparatus). The cover glass was coated with 2-hydroxyethyl methacrylate (Sigma-Aldrich, P3932) to prevent the adhesion of cardiomyocytes.

### Supporting Software

The algorithms for detrending image arrays and estimating the fundamental period of images were implemented in C for efficiency and exposed to Python using f2py (18) for efficient prototyping. The source code is available in the IOCBio Google Code project (http://iocbio.googlecode.com/). The source code of integrals of piecewise polynomial functions was generated using Sympycore (http://sympycore.googlecode.com/). To compute FFT of sequences, we used single threaded double precision FFT routines from the FFTW software library (2).

### Hardware

The tests for measuring the timings of the algorithms considered (the mean sarcomere length estimation and FFT) were performed on a Ubuntu Linux computer with a dual-core AMD Phenom(tm) II X2 550 CPU and 4 GB RAM.

## RESULTS

In this section we present the following findings of this work: *1*) conventional ACF-based sarcomere length determination methods introduce systematic errors when applied to images; the current work is providing a method that does not produce these systematic deviations. *2*) The method of current work is applied to microscopy images for determining the mean sarcomere length of a contracting cardiomyocyte. We describe the corresponding procedure, analyze sensitivity of the method to different uncertainties that are inherent to the corresponding experimental conditions, and compare the dynamics of the mean sarcomere length time evolutions obtained from different methods: the method of current work and two methods based on discrete ACF and Fourier spectrum analysis, respectively. Finally, we demonstrate the method of current work on estimating sarcomere lengths for two example experiments of cardiomyocytes.

### General Properties of Used Algorithms

#### ACF has a systematic error in sarcomere length estimation.

As a simple example, let us assume that sarcomeres induce perfectly periodic image with transmission signal on each line equal to simple sinusoidal function, *f*(*x*) = sin(2π*x*/*P*). Clearly, the period *P* corresponds to the sarcomere length. To develop methods for estimating the mean sarcomere length, *P*_est_, from an image with periodic content, we consider three approaches: *1*) the method of current work that assumes that image has strictly finite size, *2*) the conventional ACF-based method that inherently assumes that image length is made infinite by zero-padding, and *3*) the conventional periodic ACF-based method that inherently assumes that image length is made infinite by repeating the image periodically in space. (The corresponding similarity measures that define these three methods, are F^E^, F^A^, and F_periodic_^A^, respectively, all defined in appendix.) As a first example, we will estimate sarcomere length assuming that the signal is known perfectly within every pixel (a pixel carries the signal as a function rather than a single value) and the integrals corresponding to each of the methods can be found analytically. In other words, there are no artifacts induced by pixelation of the image. The defects from pixelation are demonstrated by the second example.

**Fig. 1.**
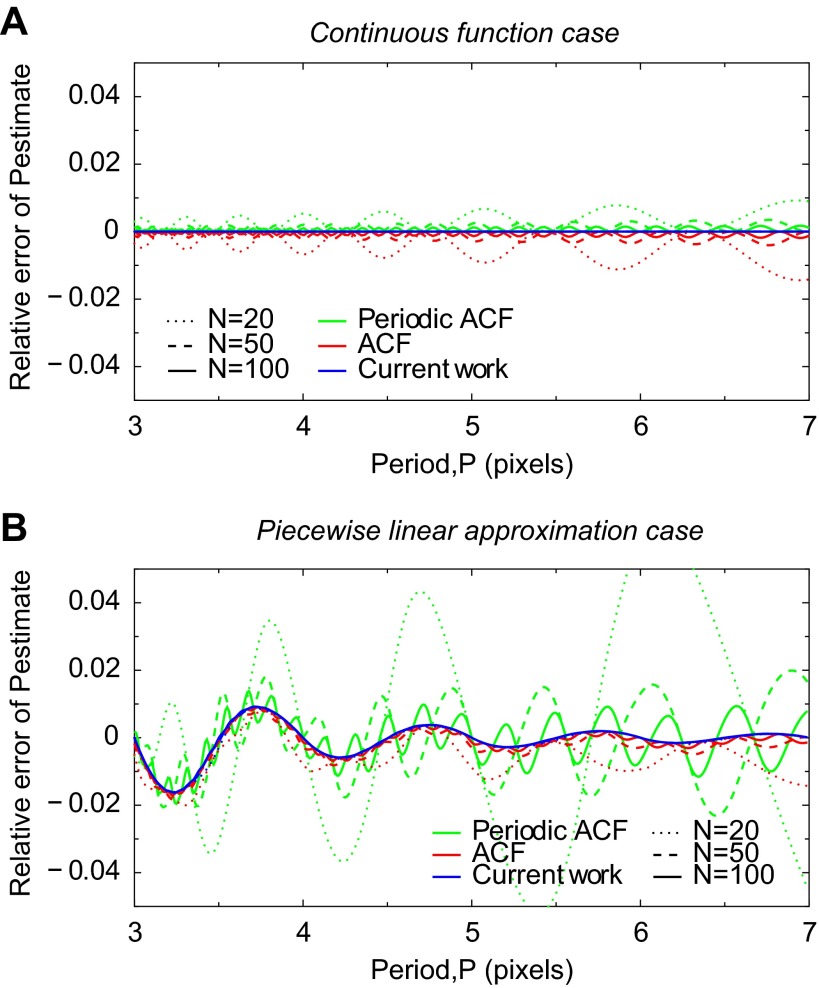
Sensitivity test of sarcomere length estimation algorithms to boundary conditions of a continuous function (*A*) and its piecewise linear approximation (*B*). Continuous function corresponds to “transmission image” (with length *N*) intensity having a form *f*(*x*) = sin(2π*x*/*P*) where *P* is the exact period and 0 ≤ *x* ≤ *N* − 1. The continuous function is then approximated with a linear spline having pixel values *f*_*i*_ = sin(2π*i*/*P*), *i* = 0; . . . . ; *N* − 1. *A* and *B* show the relative error of the period estimate *P*_est_, *P*_est_*/P* − 1, using three different methods, indicated with different line colors, and for different number of single sine waveforms per domain length, defined as the ratio *N*/*P*, and on plot indicated with different line styles. *A*: note that with *N* fixed and varying *P* the estimates predicated by the autocorrelation function (ACF) and periodic ACF analysis methods have modulated systematic errors due to imposed periodic boundary conditions: when for given *N* and *P*, the boundary values and slopes of *f*(*x*) at different ends are different, the error will be large, and when they are close, the error will be small. With the method established in this work, the estimated period is equal to the exact period for all *N* and *P* values. This indicates that this method is insensitive to the possible mismatch of boundary values at different ends of image lines. *B*: estimated periods from the all considered methods are modulated. For the method established in this work, this modulation diminishes for longer waveforms (larger *P*) because the linear approximation error will be smaller regardless of the number of waveforms per domain length (lines for different span, *N*, almost coincide). In contrast, period estimates from ACF and periodic ACF analysis methods have larger modulation amplitudes that will increase for longer waveforms. This is due to the mismatched boundary conditions as shown in *A*.

The relative errors of the period estimates are shown in Fig. 1 for three different lengths (*N*) of image lines. While the length *N* in the following example will be in pixels, here it is in arbitrary units due to the perfect image resolution used in this example. As seen on the plot Fig. 1*A*, the period estimated by ACF-based methods is modulated. The length is underestimated and overestimated, depending on the relative length of the signal period and the image line. Using the method proposed in the current work, however, always results in an exact period (relative error is identical to zero), regardless of the length of the image line or the choice of signal extension at ROI ends.

Note that the increase of modulation amplitude of *P*_est_ with the increase of *P* is directly related to the mismatch of signal at the opposing ends of the image line that all ACF-based methods inherently fail to handle. Indeed, from one hand, the number of repetitive patterns in a signal is proportional to *N*/*P*, and on the other hand, the contribution of repetitive patterns to the formation of ACF signal is proportional to the number of repetitive patterns; therefore, the relative contribution of mismatching boundary conditions to the ACF signal is greater when the number of repetitive patterns is smaller, or equivalently, *P* is larger.

Finally, we note that the modulation frequency of *P*_est_ is increasing when increasing the length of interval *N*. In linearly varying period *P*, the modulation frequency will manifest itself as plateaus in the plot of estimated period *P*_est_ (result not shown).

#### Defects from image representation by pixels.

In practice, image intensity is given at discrete points and exact analytical evaluation of similarity measures used to determine the sarcomere length (as used in Fig. 1*A*) is not possible. To be able to evaluate sarcomere length at subpixel resolution, we use linear interpolation of the signal between pixels. The inaccuracy of piecewise linear approximation, however, introduces specific errors in the estimated sarcomere length. To demonstrate this, we consider again an image represented by sinusoidal function (see above) and use its values determined on *N* pixels in an image line. Similar to the above approach, we keep the number of pixels fixed and vary *P*. Note that the corresponding integrals are evaluated using semianalytic, seminumeric approach (see appendix). The relative errors of the corresponding estimates are given in Fig. 1*B* for three different lengths of image lines. We see that the usage of linear interpolation introduces *P*_est_ oscillations around the expected sarcomere length for all considered methods.

The estimation errors of sarcomere length, manifested by the oscillations of *P*_est_, have two components. One is from mismatching boundary conditions that are the cause of increasing modulation amplitude when *P* is increased (see previous analysis on continuous signal functions). Note that this component is present, and it dominates, in the case of both ACF-based methods. The second component is due to piecewise linear approximation that has greater influence when the number of points per repetitive pattern is small, that is, when the representation of a signal function via piecewise linear interpolation is less adequate. With the increase of *P*, the errors from piecewise linear approximation decrease because the quality of piecewise linear representation increase. The outcome of this analysis is exemplified in Fig. 1*B* by the curve corresponding to the method of current work.

### Applications to Images of Cardiomyocyte

In the following we analyze different methods for estimating the mean sarcomere length of a cardiomyocyte. The methods are applied to microscopy images of a contracting cardiomyocyte that are held in microscope focus using two carbon fibers. Below we analyze in detail the case where the cardiomyocyte has no load applied other than the force generated by the elasticity of deforming carbon fibers. Finally, two example experiments with different preload and stimulation conditions on cardiomyocytes are presented.

#### Estimating the mean sarcomere length from a microscopy image of a cardiomyocyte.

**Fig. 2.**
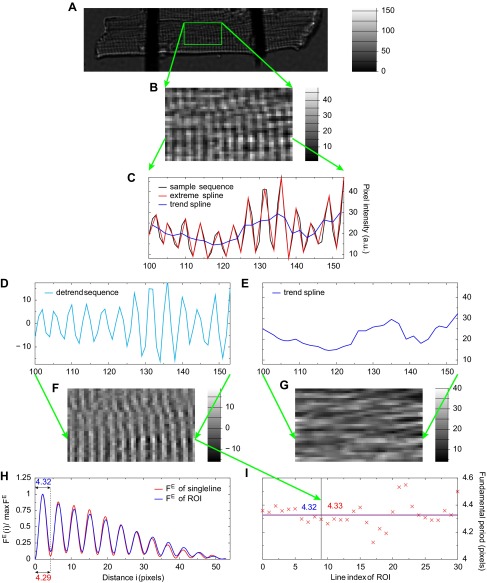
Determination of the mean sarcomere length from a regions of interest (ROI) of the microscopy image of a cardiomyocyte (*A*). The detrend algorithm is applied to each line of ROI (*B*, size is 54 × 31) that decomposes the line signal to oscillating and slowly varying parts. These parts correspond to sarcomere striation pattern and uneven background, respectively. *C*-*E*: decomposition of the 10th line of the ROI. *F* and *G*: results of the detrend algorithm applied to all lines in the ROI image. Next, the fundamental period (i.e., sarcomere length) of the sarcomere striation pattern is estimated as the minimum point of the similarity measure (F^E^) shown in *H* for the 10th line and for the whole ROI. *I*: illustrates how the sarcomere length estimates from single lines (red crosses, average is 4.33) relate to the estimate from the ROI image (blue level line at 4.32).

The procedure of estimating the mean sarcomere length of a cardiomyocyte is exemplified in Fig. 2 and explained as follows. The procedure starts with acquiring a transmission image of a cardiomyocyte (Fig. 2*A*) and selecting a ROI (Fig. 2*B*) for subsequent image preprocessing step. The aim of the preprocessing step is to discard any nonperiodic content from the ROI image. For that we use the detrend algorithm (Fig. 2*C*, see appendix for detailed description) that decomposes each line of the ROI image into oscillatory part (Fig. 2*D*) and slowly varying part (Fig. 2*E*). The image analysis continues by applying the sarcomere length estimation algorithm to the oscillatory part of the ROI image (Fig. 2*F*; the slowly varying part is discarded but is shown in Fig. 2*G* for illustration purposes). As explained in detail in appendix, the sarcomere length estimate is defined as the first positive minimum point of a similarity measure; here we use F^E^ that is similarity measure proposed by the current work and that turns out to be most appropriate for images. When estimating sarcomere length from multiple image lines, such as selected ROI, two approaches are possible: *1*) compute the similarity measure of ROI image as the sum of similarity measures of each image line and then find the minimum point of the similarity measure or *2*) find the minimum points of similarity measures computed for each line and then compute the mean minimum point. Figure 2, *H* and *I*, illustrates that the two approaches lead to slightly different results. In this particular case, the approach with the superposition of similarity measures of ROI lines (blue line in Fig. 2*H*) leads to a sarcomere length equal to 4.32 pixels, while the average of all minimum points (red crosses in Fig. 2*I*) of similarity measures is 4.33 pixels. For the rest of this study, we choose to use the first approach as it appears to be more robust in practice. For instance, it is possible that a few lines in a ROI image contain no sarcomere striation pattern information, and therefore, the estimation of fundamental period on those lines is impossible and the second approach becomes less reliable. Finally, taking into account the pixel size of the microscopy image, we obtained the mean sarcomere length. In our microscope setup, the pixel size is 0.411 μm, and therefore, the mean sarcomere length is estimated to be 4.32 × 0.411 μm ≈ 1.775 μm for the given time moment of cardiomyocyte contraction.

#### Sensitivity analysis of the choice of ROI.

In practice, the results of the mean sarcomere length estimation procedure (Fig. 2) may strongly depend on the choice of ROI either because of heterogeneity of sarcomere striation patterns within a single cardiomyocyte or because of various experimental conditions that may influence the visibility of sarcomeres. Therefore, understanding the sensitivity of the choice of ROI is important when selecting the ROI for sarcomere length analysis. In general, the choice of ROI is characterized by its location, size, and orientation with respect to the original image. In the following we analyze how each parameter of ROI influences the estimation of the mean sarcomere length when applied to the same microscopy images of a cardiomyocyte. Due to extensive computations, use of full sensitivity analysis in real time is probably impossible, unless some specialized computational techniques are utilized.

**Fig. 3.**
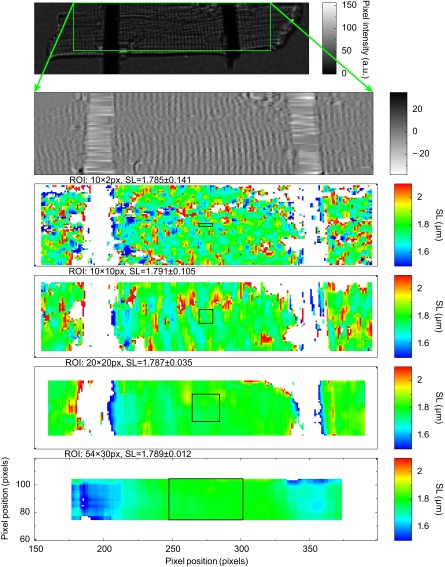
Sensitivity analysis of sarcomere length (SL) estimation to ROI size and location. A periodic content of a region in a transmission image of a cardiomyocyte is found (*top two plots*). The mean sarcomere length is computed for each point of the region using different ROI sizes (*bottom four plots*). The ROI size used is illustrated with a black box. White pixels denote regions where the sarcomere length could not be computed due to a lack of sarcomere signal or due to the presence of carbon fibers. Each pixel value, indicated by color bar at *right*, corresponds to the mean sarcomere length estimate obtained from a ROI that center is located at the given pixel position and that has specified size. For small ROIs, heterogeneity of sarcomere length within the cardiomyocyte is notable (*top two color plots*). Usage of larger ROIs provides the mean sarcomere length that is insensitive to the position of the ROI (*bottom two color plots*).

Figure 3 shows how the mean sarcomere length estimate depends on the size of ROI and its location in an overall image. Usage of smaller ROI sizes reveals a slight heterogeneity of the mean sarcomere length within a cardiomyocyte microscopy image. This heterogeneity can be explained by the heterogeneous morphology of cardiomyocyte that partly appears in the form of dislocations of the repeating patterns in the microscopy image. With larger ROI sizes, this heterogeneity is averaged out and the mean sarcomere length is stable with respect to the choice of ROI location, even in the proximity of attached carbon fibers. On the other hand, a selection of larger ROI increases the computational requirements of the mean sarcomere length estimation, although this increase has no practical influence as the typical acquisition frame time is an order of magnitude larger than the typical time of sarcomere length estimation [for a typical 54 × 31 (length × height) ROI size it takes ∼16 ns to estimate the mean sarcomere length on our computer platform].

**Fig. 4.**
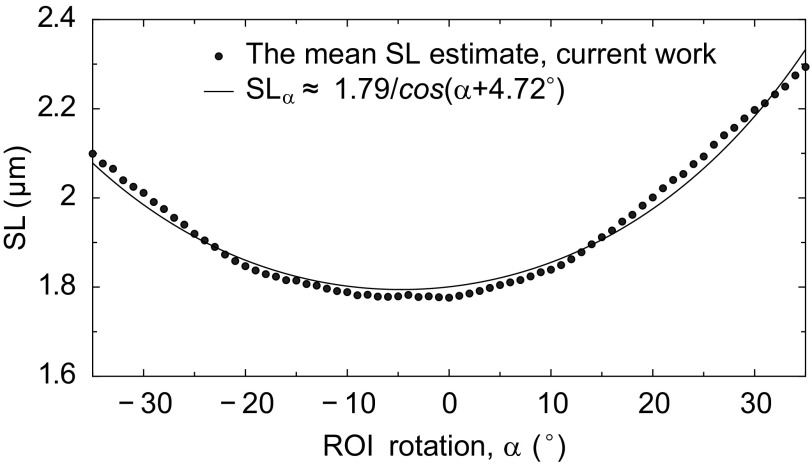
Sensitivity analysis of the mean sarcomere length estimation to the selection of ROI orientation. The mean sarcomere length estimate is inversely proportional to the cosine of ROI orientation angle. The minimum point corresponds to ROI orientation that coincides with the orientation of sarcomeres and, hence, leads to a corrected estimate of the mean sarcomere length.

Figure 4 illustrates how different ROI orientations affect the result of the mean sarcomere length estimation. Because the original image data are given on a rectangular grid, we use bilinear interpolation to compute the ROI image data on the rotated rectangular grid. Notice that the mean sarcomere length estimate is approximately inversely proportional to the cosine of ROI orientation angle. This follows from a general consideration of cutting a *P*-periodic lattice of lines at the orientation angle α leading to the formula *P*/cos(α − α_0_) that defines the period of the cut structure. Here α_0_ corresponds to the optimal orientation of the periodic lattice. In other words, by estimating sarcomere length from ROI where the sarcomeres are not aligned along the lines, the estimated sarcomere length is by 1/cos(α − α_0_) larger than the length corresponding to the cardiomyocyte. Such analysis could be used to eliminate the small deviation in the mean sarcomere length estimate due to the imperfect choice of ROI orientation. In the given example, the optimal orientation of ROI is found to be at the angle −4.72° and the improved estimate to the mean sarcomere length is obtained by multiplying the original estimate of the mean sarcomere length with a correction factor cos 4.72° ≈ 0.997. Below we also report how the contraction of a cardiomyocyte affects the optimal orientation of a ROI and how the estimate of the mean sarcomere length is influenced by that.

#### Time evolution of the mean sarcomere length in a contracting cardiomyocyte.

**Fig. 5.**
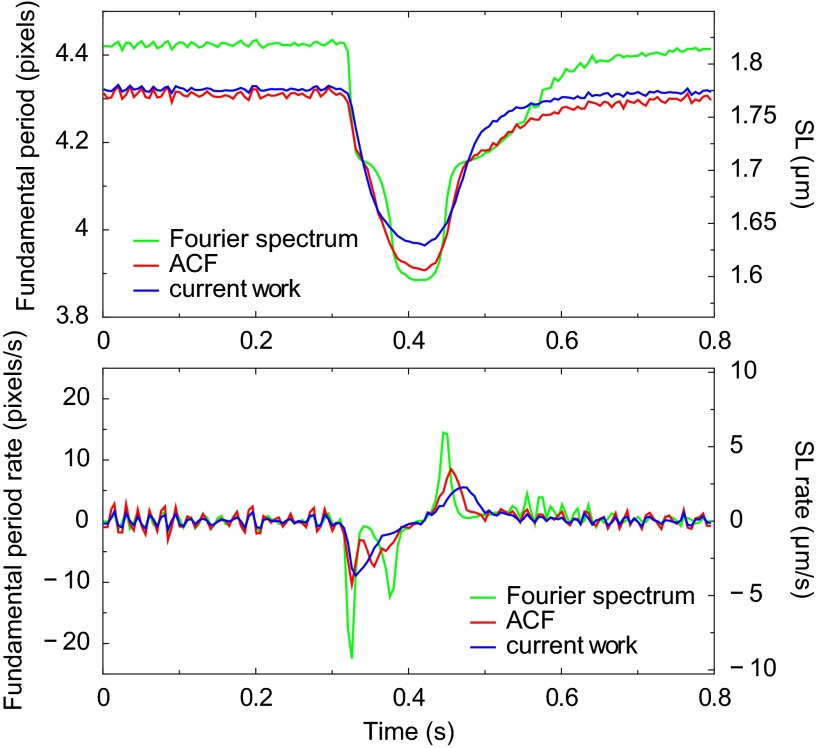
Determination of sarcomere length in a contracting rat cardiomyocyte. *Top*: time evolution of the mean sarcomere length estimated by three methods. Microscopy images of a cardiomyocyte are recorded at rate 200 Hz and the same ROI, as in Fig. 2, is used. The methods based on Fourier spectrum and ACF analysis clearly demonstrate modulation defects: plateaus in the mean sarcomere length curve and spurious peaks in the mean sarcomere length rate curve (*bottom*). The mean sarcomere length estimate from the method of current work does not have these artifacts.

The time evolution of the mean sarcomere length estimate during one cardiomyocyte contractions is shown in Fig. 5. The plot is produced from CCD camera images recorded at 200 Hz (time resolution is 5 ms). At *top*, the mean sarcomere length estimates from three different methods are shown. The ROI size in all cases is 54 × 31.

First, the method based on Fourier spectrum analysis provides worst estimates as during the evolution of the mean sarcomere length smooth plateaus appear. When selecting a longer ROI with more sarcomeres in the image line, the steps between the plateau levels will the decrease (result not shown for brevity). Note that this behavior is manifestation of systematic modulation frequency in sarcomere length estimation methods that are based on Fourier spectrum analysis (see Fig. 1*A*). Therefore, to suppress these artificial plateaus, one should increase the length of a ROI.

Second, when using the quadratic approximation of averaged ACF of ROI image lines, the phenomenon of plateaus in sarcomere length evolution is somewhat reduced but still notable. We suspect that these plateaus have the same origin as in the method based of Fourier spectrum analysis.

Third, the method developed in the current work (see appendix) gives sarcomere length estimates with no apparent artifacts other than small noise. Notice that the mean sarcomere length estimates differ from each other up to 2.5% in both directions. For instance, at the resting state the Fourier spectrum estimate is ∼2.5% larger than the estimates from methods using ACF or of current work; at the maximal contraction moment it is ∼2.5% smaller than the estimate from the method of current work. This difference is in agreement with the analysis of estimating the period of a discrete sine function where the relative error of the estimates can be up to 2% (in Fig. 1*B*, consider the curve corresponding to the periodic ACF-based method and *N* = 50, which is closest to considered ROI image length 54; the relative error is estimated around the point 4.3). Therefore, we conclude that the differences in the mean sarcomere length estimates are due to *1*) boundary artifacts and *2*) piecewise linear interpolation approximation of the intensity field. The former dominates the latter in the case of Fourier spectrum and ACF analysis-based methods, while for the method of current work it is not present, and only the latter can cause relative errors up to 1%. We note that estimating the relative errors of the mean sarcomere length based on Fig. 1*B* is plausible if the amplitude of the signal is constant, as in the artificial examples in Fig. 1. Otherwise, the amplitude variations such as demonstrated in Fig. 2*D*, contribute to the uncertainty of the mean sarcomere length estimates.

Figure 5, *bottom*, shows the rate estimates of the mean sarcomere length (computed as 3-point finite difference of the corresponding sarcomere length sequences) for each of the three methods. Here, the plateau artifacts from spectral and ACF-based methods appear as spurious peaks in the sarcomere length rate evolution. In summary, Fig. 5, *A* and *B*, demonstrates the inherit problems in using spectral and ACF approaches and the reliability of our method for estimating the mean sarcomere length and its rate.

**Fig. 6.**
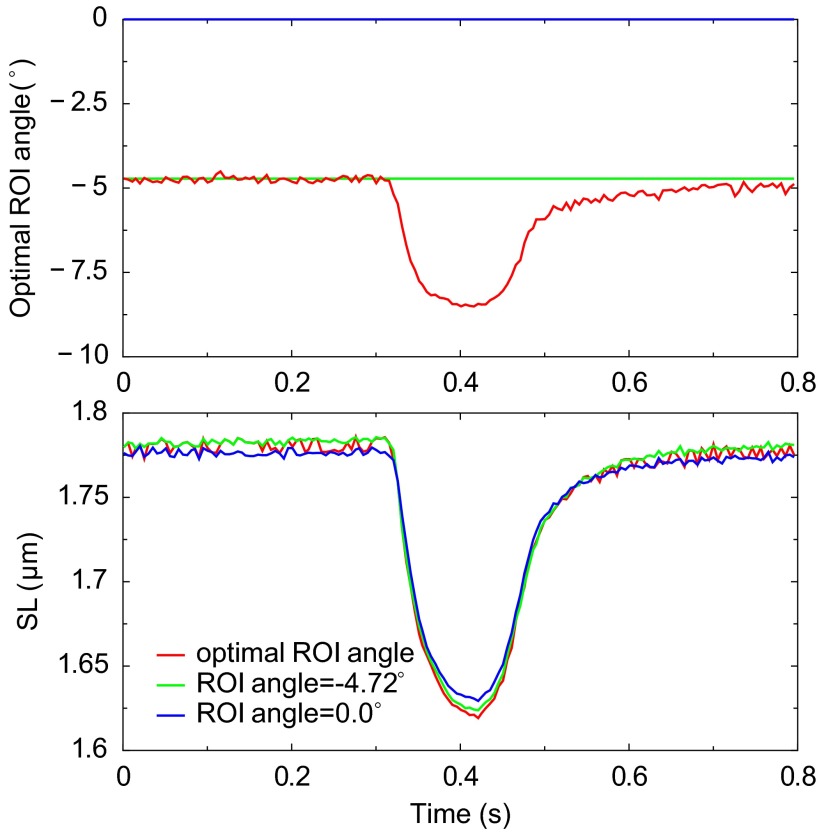
Correction of ROI orientation in the determination of mean sarcomere length in a contracting rat cardiomyocyte. *Top*: orientation of ROI as a function of time. The optimal ROI orientation is defined as the average orientation of sarcomere striation pattern that is determined form the sarcomere length sensitivity analysis to ROI selection (see Fig. 4 and results). ROI angle −4.72° corresponds to the optimal ROI orientation of the first image where cardiomyocyte is at relaxed state. ROI angle 0° corresponds to initial ROI orientation selected visually by operator. *Bottom*: mean sarcomere length estimates computed from the three ROI orientation selections where one is time dependent. Notice that the mean sarcomere length estimate with no ROI orientation correction is rather close to the estimates that take into account the cardiomyocyte orientation variations during contraction.

Finally, we note that the orientation of sarcomeres may change during the contraction of a cardiomyocyte. This orientation change can be determined using a sensitivity analysis of sarcomere length to ROI orientation (see Fig. 4) applied to each microscopy image of the cardiomyocyte, although not in real time due to extensive computational requirements. Figure 6, *top*, shows the evolution of an optimal ROI angle, or equivalently, the average orientation angle of sarcomeres during the contraction. Figure 6, *bottom*, shows the evolution of the estimated mean sarcomere length, accompanied with two sarcomere length estimates from correcting the ROI orientation with two methods: *1*) using the time-dependent optimal ROI angle and *2*) using fixed optimal ROI angle from the first microscopy image. Clearly, the mismatch of ROI orientation to the variable sarcomere orientation has only a small effect on the mean sarcomere length estimates when comparing with the case without orientation correction (ROI angle = 0°).

#### Invariance of the mean sarcomere length to the ROI selection in the case of a homogeneous contraction of cardiomyocytes.

We have analyzed the sensitivity of the mean sarcomere length estimate to ROI size and location at different stages of cardiomyocyte contraction: at the resting stage *t* = 0 *s* (in Fig. 3 for largest considered ROI size the mean sarcomere length over a region between carbon fibers is SL = 1.789 ± 0.012, using the method of current work on all possible ROIs between carbon fibers), at the beginning of contraction *t* ≈ 0.32 s (SL = 1.784 ± 0.010), at the most rapid stage of contraction *t* ≈ 0.34 s (SL = 1.709 ± 0.007), at the shortest sarcomere length stage *t* ≈ 0.42 s (SL = 1.640 ± 0.008), and at the relaxation stage *t* ≈ 0.48 s (SL = 1.715 ± 0.009, all time points present in Fig. 5). For small ROI sizes that resolve the small heterogeneity of sarcomeres within the cardiomyocyte we have not observed that certain regions would contract more rapidly than others (results not shown). From these results we conclude that the contraction is uniform over a particular cardiomyocyte (at the acquisition rate of 200 Hz) and the mean sarcomere length estimate does not depend on the position of ROI even during the contraction. This conclusion is in agreement with the results from Gannier et al. (3). Note, however, our study is performed on cardiomyocyte images where nuclei are far from the focal plane, and therefore, no signs of significant sarcomere length variations were detected. Although in the proximity of nuclei, the variations of sarcomere length could be expected (19). Also note that the cardiomyocyte under study is almost unloaded; only the elasticity of fibers load the cardiomyocyte during contraction.

#### Example experiments.

**Fig. 7.**
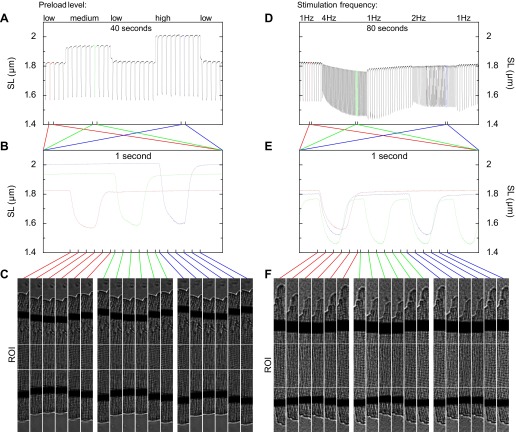
Determination of sarcomere lengths in single rat cardiomyocyte experiments with different preload levels (*left*) and different stimulation frequencies (*right*). *A* and *D*: time series of sarcomere lengths over experiment periods 40 and 80 s, respectively. During the experiment periods the preload and stimulation conditions are varied. *B* and *E*: sarcomere lengths over a 1-s period as recorded during different loading conditions. *C* and *F*: transmission images of cardiomyocytes at time moments indicated with connecting lines between the *B* and *E* and *C* and *F*. The region between lines of white pixels indicates the ROI that is used for determining the mean sarcomere lengths for each captured image of cardiomyocytes.

In the following we provide two examples of experiments on single cardiomyocytes that illustrate the response of sarcomere length estimates to changing preload conditions and to switching stimulation frequencies, both represented in Fig. 7. Increasing the preload level of a cardiomyocyte by two attached carbon fibers leads to an increase of contraction extent as seen on the time series of the mean sarcomere length. This is a manifestation of the Frank-Starling law at a single cardiomyocyte sarcomere level. Switching stimulation frequency also leads to changes in contraction extent but with a longer transition period characterizing the adoption of cardiomyocyte to new loading conditions.

## DISCUSSION

In this work we developed a computationally efficient and practical method for estimating the mean sarcomere lengths of a single contracting cardiomyocyte. Input to the method is a microscopy image of a cardiomyocyte (or ROI image) that is assumed to contain sarcomere striation patterns. Uneven background due to possibly visible nuclei, external carbon fibers, or otherwise unfavorable optical conditions is filtered out in image preprocessing step by using the introduced detrend algorithm. The preprocessed image is compared with its own shifted copy to find the smallest nonzero shift at which the two images have the closest match. We define a similarity measure to quantify such a match. Since the optimal shift is a continuous parameter as opposed to the grid of microscopy image, we use piecewise linear approximation of the image to achieve the estimate of the mean sarcomere length at subpixel resolution. While conventional methods based on Fourier spectrum and ACF analysis could use a similar approach, our method takes into account the fact that microscopy images have finite extent, and as a result, the mean sarcomere length estimates are not artificially modulated by boundary conditions that are the main source of systematic errors in the mentioned conventional methods. We have performed the sensitivity analysis of the proposed method to uncertainties such as ROI size, location, and orientation that are all common when executing experimental protocols on contracting cardiomyocytes that are controlled, for instance, by attached carbon fibers.

Measurement of the mean sarcomere length is essential for many experimental protocols. For example, as a prerequisite of carrying out experiments that use cardiomyocytes, the quality of the cell must be assessed. The cell quality can be characterized by the size of diastolic sarcomere length as well as by the extent of cardiomyocyte contraction that is quantified via sarcomere length. Sarcomere length of a cardiomyocyte can be determined via microscope imaging and using specialized image processing technique, such as one developed in this study. Measurement of sarcomere length is crucial for experimental protocols that measure the mechanical properties of cardiomyocytes. For example, cardiomyocyte mechanical properties are studied by determining the force and length relationship of cardiomyocyte under a variety of mechanical conditions such as isometric and physiological conditions (16, 5) or by determining differences in the contractile properties of control and diseased animal models (15). In addition, cell stretching is used to study the mechanoelectrical feedback of cardiomyocyte where recording of sarcomere length is important (14, 6). Real-time measurement of sarcomere length has also been used for measuring muscle performance in vivo (13) to investigate neuromuscular disorders.

In the present study we developed a method for estimating the mean sarcomere length from microscopy images obtained with a high-speed camera capturing transmission light. The same method could be applied to fluorescence microscopy images where sarcomere locations are revealed with quantum dots (21) or using, for instance, two-photon excitation microscopy (1). Other approaches exists for estimating the mean sarcomere length of cardiomyocytes. For instance, the analysis of X-ray or laser-light diffraction patterns gives accurate sarcomere length estimates on both the muscle fiber level (12, 7) and cell level (9, 11). With the advances in CCD/CMOS technology and image processing techniques, the analysis of microscopy images from high-speed cameras provides alternative techniques to diffraction-based methods with potentially similar accuracy and performance properties, while at the same time being simple and cost-effective.

The mean sarcomere length is defined as the spatial fundamental period of repeating patterns of sarcomeres in a microscopy image of a cardiomyocyte. The technique developed in this study for estimating the mean sarcomere length uses a number of novel approaches. *1*) From the inherit reality of measuring signals with finite length, the underlying mathematical theory for the sarcomere length estimation technique considers functions on finite interval and proposes an unbiased measure of similarities for a pair of functions. We have shown that the ACF, which provides a popular measure of similarities due to its efficient evaluation via FFT, is biased: for otherwise uniformly changing sarcomere length, the sarcomere length estimate from ACF analysis is always modulated. In addition, because FFT-based methods impose periodic boundary conditions to otherwise finite signal sequences, the modulations of sarcomere length estimates are then amplified even more due to the mismatch between the period imposed by FFT to the overall signal and the fundamental period, such as sarcomere length, within the signal. Although practical tricks exist to reduce this modulation defect, in our approach, however, these artificial constraints are avoided. *2*) Our technique uses a semianalytical, seminumerical approach for evaluating the measures of similarities efficiently. With the use of a piecewise linear representation of otherwise discrete signals, the integrals in similarity measures are resolved analytically. As a result, the similarity measure is a piecewise cubic polynomial whose nodal values are a finite series of signal samples. From that, the mean sarcomere length is estimated analytically as the minimum point of one of the cubic polynomials in this piecewise polynomial representation of the similarity measure. Such an approach enabled us to implement a numerical algorithm for the mean sarcomere length estimation that has superior computational performance to FFT-based methods. *3*) We have introduced an image preprocessing detrend algorithm that extracts periodic variations from a microscopy image with possibly nonuniform background. This algorithm can be used in combination with ACF-based methods where preprocessing of images to a zero-average condition is also needed. As discussed below, the detrend algorithm improves the stability of sarcomere length estimates in experiments.

Our sarcomere length estimation algorithm is closely related to ACF-based algorithms for determining fundamental periods in images. In fact, if the image of sarcomeres would be perfectly periodic, then both methods would return exactly the same estimates. ACF-based algorithms can take advantage of both the detrend algorithm and the used approach to achieve estimates at subpixel resolution. However, when the image has limited size, as it is common in practice, the estimate from a ACF-based algorithm is biased due to the mismatch of image intensities at the boundaries: the maximum point of ACF, which determines the estimated value, is slightly shifted from the exact value. Depending on how the image of sarcomeres is cut off at boundaries, the estimate can be either smaller or larger than the exact value. The extent of this deviation will decrease when longer sarcomere sequences are included to the image, say, by increasing the length of ROI. In contrast, the method developed in this work is unbiased to the image boundary conditions.

To summarize, the accuracy of the developed method is limited only by the following practical restrictions: *1*) the subpixel accuracy of the mean sarcomere length estimate is limited by the finite image resolution, and *2*) apparent inhomogeneous distribution of sarcomeres, both in length and orientation, will increase the uncertainty of sarcomere length estimates and makes introduction of the notation of the mean sarcomere length unavoidable. In theory, our method determines the fundamental period exactly when the object field has a unique periodic component and finite size. Thus our method circumvents one of the practical limitations of conventional methods based on ACF or Fourier spectrum analysis. With the use of the conventional methods, the mean sarcomere length estimate will always be artificially modulated during the contraction of cardiomyocyte because of the finite size of microscopy images.

The technique developed in this study has been successfully tested on microscopy images of rat cardiomyocytes. The sarcomere length sensitivity analysis to ROI selection showed that the method is robust to a variety of experimental constraints such as uneven field of illumination (resolved by the detrend algorithm), existence of external objects (such as carbon fibers) in the field of view, and uncertainty of choosing the orientation of ROI with respect to variable orientation of sarcomeres (also during contraction). While the speed of sarcomere length estimation was more than enough for our experimental protocols (maximal line rate of our CCD camera is ∼1 kHz while the rate of sarcomere length estimation with full 120 × 60 ROI in between carbon fibers was ∼15 kHz), with recent and future advances in high-speed camera technologies and with larger ROI selections one could optimize the sarcomere length estimation rate even further, for example, by implementing the sarcomere length estimation algorithm in a field-programmable gate array chip. According to our tests, a more than doubled rate of sarcomere length determination is achieved when disabling the detrend algorithm. This, however, requires that images have an uniform background field and no external objects such as carbon fibers exist within the ROI. Another approach would be to use a parallelized implementation of the proposed algorithm.

To obtain meaningful sarcomere length estimates, selection of computational methods and ROI positions is of importance. In this study, we showed that ACF and Fourier spectrum analysis-based methods modulate the estimates of the sarcomere length considerably when the number of sarcomeres per ROI length is relatively small. The method provided by the current work eliminates this modulation defect. We have also showed that the misalignment of ROI orientation to sarcomere striation pattern will produce overestimates to the mean sarcomere length. The location of ROI within the cardiomyocyte image is also important. Depending on the used attachment techniques of cardiomyocyte ends and applied loading (17, 16, 5), ROI should be placed to location where sarcomere striation pattern is uniform and as far as possible from attachment points. When using the carbon fiber attachment technique, the distribution of sarcomeres is found to be fairly homogeneous between carbon fibers as well as between different sides of a cardiomyocyte (5). The size of ROI should be as large as possible to increase the amount of information on the periodic variations of sarcomere striation patterns but not larger than the local uniformity of the striation patterns would allow.

In summary, the developed algorithm for the mean sarcomere length estimation is proposed as a superior algorithm to popular ACF and spectral analysis-based sarcomere length estimation techniques. In fact, the developed algorithm can be used for determining the fundamental periods of any signals, and hence, the algorithm has much wider applications. The C and Python source code of developed methods are available in the IOCBio Google Code project (http://iocbio.googlecode.com/) under the Open Source Initiative BSD-2 License.

## GRANTS

This work was supported by the Wellcome Trust Fellowship No. WT081755 and Estonian Science Foundation Grant No. 7344 and 8041 (PhD stipend to M. Kalda).

## DISCLOSURES

No conflicts of interest, financial or otherwise, are declared by the author(s).

## AUTHOR CONTRIBUTIONS

Author contributions: P.P., M.K., and M.V. conception and design of research; P.P. analyzed data; P.P., M.K., and M.V. interpreted results of experiments; P.P. prepared figures; P.P. drafted manuscript; P.P., M.K., and M.V. edited and revised manuscript; P.P., M.K., and M.V. approved final version of manuscript; M.K. performed experiments.

## Appendix

Here, a mathematically rigor description of the algorithms is given.

### Similarity Measures

To find the mean sarcomere length of a cardiomyocyte, we use image and its shifted copy. The mean sarcomere length is assumed to correspond to the shift that leads to the smallest difference, i.e., largest similarity, of the image and its shifted copy. In this section we define three possible functionals that measure similarity of a given function and its shifted counterpart. These functionals are used as objective functions to a minimization problem that solution corresponds to the fundamental period of the given function.

First, as a basic tool of the current work, we define a similarity measure between the function *f*(*x*) and its shifted counterpart *f*(*x* + *y*) as follows:

(1) $${\text{F}}^{\text{E}}\left[f\right]\left(y\right)=\int {\left[f\left(x+y\right)-f\left(x\right)\right]}^{2}dx,$$

where the integration interval depends on the type of functions: for periodic functions the integration is carried out over an interval with the length of a period and for functions on a finite interval over a subinterval where *f*(*x* + *y*) and *f*(*x*) are defined simultaneously. Clearly, the measure F^E^ has a local minimum at the fundamental period and we can use it as objective function for finding the fundamental period estimate of a quasiperiodic function *f* = *f*(*x*): *P*_est_^E^[*f*] = argmin_*y*>0_ F^E^[*f*](*y*), where *P* = argmin_*y*>0_ *F*(*y*) means that *P* is a positive minimum point of a function *F*(*y*).

Expansion of the integrand in *Eq. 1* yields

(2) $${\text{F}}^{\text{E}}\left[f\right]\left(y\right)=\int f{\left(x+y\right)}^{2}dx+\int f{\left(x\right)}^{2}dx-2\text{ACF}\left[f\right]\left(y\right),$$

where

(3) ACF[f](y)=∫f(x)f(x+y)dx

is the ACF of *f*(*x*). For periodic functions, the first two terms in *Eq. 2* are shift variable *y* independent, and hence, all local minima of F^E^ coincide with the corresponding local maxima of the ACF. For functions on finite interval, however, the locations of these minima and maxima are different because all three terms in *Eq. 2* depend on the shift variable. Hence, functional ACF cannot be used as an unbiased measure of similarities for functions on finite interval, although it is unbiased measure for periodic functions. The functional F^E^, however, is an unbiased measure for all considered types of functions.

To demonstrate how biased measures, such as ACF, influence the accuracy of estimated fundamental period when functions are defined on finite interval, we define an ACF-based functional

(4) $${\text{F}}^{\text{A}}\left[f\right]\left(y\right)=-\int_{0}^{L-y}f\left(x\right)f\left(x+y\right)dx,$$

and the corresponding fundamental period estimate: *P*_est_^A^[*f*] = argmin_*y*>0_ FA[*f*](*y*). To demonstrate the same with the class of periodic functions, we define

(5) $${\text{F}}_{\text{periodic}}^{\text{A}}\left[f\right]\left(y\right)=-\int_{0}^{L-y}f\left(x\right)f\left(x+y\right)dx\;-\int_{L-y}^{L}f\left(x\right)f\left(x+y-L\right)dx.$$

Note that this functional is equivalent to the conventional ACF of periodic functions that can be efficiently computed using the convolution theorem and FFT algorithm.

### Semianalytical, Seminumerical Evaluation of Similarity Measures

Given a signal as a sequence of signal values

$$f={\left\{f_{i}\right\}}_{i=0}^{N-1}$$

, we define a piecewise polynomial function, a so-called signal function, as follows:

(6) $$f\left(x\right)=\overset{N-2}{\underset{i=0}{\sum }}\left[0\leq x-i<1\right]S\left(x-i;\text{f},i\right),$$

where *S* denotes a low order polynomial defined for the interval [*I*, *i* + 1] such that *S*(0; **f**, *i*) = *f*_*i*_ with additional continuity conditions on the derivatives of *f*(*x*) when possible; *S* can be a basic spline function, for instance. The square brackets in *Eq. 6* denote an indicator function: when the condition within the brackets is satisfied then the value of the indicator function is 1; otherwise 0. The signal function *f*(*x*) is defined on the interval [0, *L*] where the length of the interval, *L*, is related to the number of signal values *N*: *L* = *N* − 1. In this study, we use linear polynomials for *S* because then the minimum points of considered functionals may locate within integer intervals, which is a prerequisite for resolving fundamental periods at subpixel resolution. In addition, the minimum conditions are quadratic equations that are easy to solve.

To evaluate the integral in similarity measure *Eq. 1*, we use the following general result: if *G*[*f*(*x*), *f*(*x* + *y*)] is a polynomial function of signal functions *f*(*x*) and *f*(*x* + *y*), then

(7) $$\int_{0}^{L-y}G\left[f\left(x\right),f\left(x+y\right)\right]dx=\overset{N-2-\left\lfloor y\right\rfloor }{\underset{i=0}{\sum }}\int_{0}^{1-\left\{y\right\}}G\left[S\left(s;\text{f},i\right),S\left(s+\left\{y\right\};\text{f},i+\left\lfloor y\right\rfloor \right)\right]ds+\overset{N-3-\left\lfloor y\right\rfloor }{\underset{i=0}{\sum }}\int_{1-\left\{y\right\}}^{1}G\left[S\left(s;\text{f},i\right),S\left(s+\left\{y\right\}-1;\text{f},i+\left\lfloor y\right\rfloor +1\right)\right]ds,$$

where ⌊y⌋ and {*y*} denote integer floor and remainder of *y*, respectively, such that *y* = ⌊y⌋ + {*y*}. When evaluating the integrals in *Eq. 7* analytically, the result will be a polynomial in {*y*}with coefficients expressed as series depending on ⌊y⌋. For example, for linear polynomials, *S*(*s*; **f**, *i*) = (1 − *s*)*f*_*i*_ + *sf*_*i*+1_, the integral in *Eq. 1* can be evaluated using

(8) $${\text{F}}^{\text{E}}\left[f\right]\left(y\right)=e_{0}\left(\left\lfloor y\right\rfloor \right)/3+e_{1}\left(\left\lfloor y\right\rfloor \right)\left\{y\right\}+e_{2}\left(\left\lfloor y\right\rfloor \right){\left\{y\right\}}^{2}+e_{3}\left(\left\lfloor y\right\rfloor \right)/3{\left\{y\right\}}^{3},$$

where

$$e_{0}\left(j\right)=f_{N-2}^{2}+f_{N-1}\left(f_{N-1}+f_{N-2}\right)+f_{N-j-1}\left(f_{N-j-1}-f_{N-2}\;-2f_{N-1}\right)+f_{N-j-2}\left(f_{N-j-1}+f_{N-j-2}-f_{N-1}-2f_{N-2}\right)\;+\overset{N-3-j}{\underset{i=0}{\sum }}f_{i}\left(f_{i}-f_{i+j+1}-2f_{i+j}\right)+f_{i+1}\left(f_{i+1}+f_{i}-f_{i+j}-2f_{i+j+1}\right)\;+f_{i+j}\left(f_{i+j+1}+f_{i+j}\right)+f_{i+j+1}^{2},$$

$$e_{1}\left(j\right)=+f_{N-2}\left(f_{N-j-2}-f_{N-2}\right)-f_{N-j-2}f_{N-1}+f_{N-j-1}\left(f_{N-2}+f_{N-1}-f_{N-j-1}\right)+\sum_{i=0}^{N-3-j}f_{i+j}\left(f_{i+1}+f_{i}-f_{i+j}\right)+f_{i+j+1}\left(f_{i+j+1}-f_{i+1}-f_{i}\right),$$

$$e_{2}\left(j\right)=+f_{N-2}\left(f_{N-2}-f_{N-1}\right)+f_{N-j-2}f_{N-1}+f_{N-j-1}\left(f_{N-j-1}-f_{N-j-2}-f_{N-2}\right)+\sum_{i=0}^{N-3-j}f_{i+j}^{2}+\left(-f_{i+j}-f_{i+j+2}\right)f_{i+1}+f_{i+j+1}\left(2f_{i+1}+f_{i+j+2}-f_{i+j}-f_{i+j+1}\right),$$

$$e_{3}\left(j\right)=-f_{N-1}^{2}+f_{N-2}\left(2f_{N-1}-f_{N-2}\right)+f_{N-j-2}\left(f_{N-1}-f_{N-j-2}\;-f_{N-2}\right)+f_{N-j-1}\left(2f_{N-j-2}+f_{N-2}-f_{N-j-1}-f_{N-1}\right)\;+\overset{N-3-j}{\underset{i=0}{\sum }}f_{i+j+2}\left(f_{i+1}+f_{i+j+2}-f_{i}\right)+f_{i+j}\left(f_{i+1}-f_{i}-f_{i+j}\right)\;+2f_{i+j+1}\left(f_{i}+f_{i+j}-f_{i+1}-f_{i+j+2}\right).$$

Similar expressions can be derived for F^A^[*f*] and F_periodic_^A^[*f*]. The corresponding computational routines are provided in *Supporting Software*.

### Determination of the Fundamental Period

In this work, the fundamental period of a signal is estimated as the first positive minimum point of a similarity measure (F^E^, F^A^, and F_periodic_^A^). By introducing a piecewise linear signal function, the similarity measure is a piecewise cubic polynomial and its minimum point is found as the second positive zero point of its derivative (the first one corresponds to local maxima). In general, the computational complexity of finding the fundamental period of a signal is

O[(N−⌊P⌋)⌊P⌋]

, where *N* and *P* are the length and the fundamental period of the signal, respectively. Compare this to the computational complexity *O*(*N* log *N*) of the FFT algorithm.

In the case of a microscopy image that is represented as a two-dimensional array of signal sequences, we define an average similarity measure computed as the average of similarity measures of all lines in the image ROI. The fundamental period estimate of the image array is defined as the first positive minimum point of the average similarity measure. Alternatively, one could compute the fundamental period estimate for each line of the image ROI and then by averaging over all lines find the mean fundamental period estimate. In general, the estimates of both approaches will be slightly different. We prefer the first one because it turns out to be more robust to images where sarcomere related intensity variations are weak.

### Detrending a Signal

The detrend algorithm consists of four steps. In the first step, random noise in a signal sequence is suppressed using convolution with a uniform kernel that has a half-width of ∼1/10 of the fundamental period. In the second step, all local extrema are found from the obtained noise-suppressed sequence. The graph of these local extrema are connected with line segments constituting a linear spline that we denote as an extreme spline. In the third step, the middle points of the line segments of the extreme spline are connected with lines. The result constitutes another linear spline that we denote as a trend spline. Finally, the oscillatory part of the signal sequence (including random noise) is obtained by subtracting the trend spline from the original signal sequence. This method of extracting the oscillatory part of a sequence is implemented in a computer program using a single loop and thus has computational complexity *O*(*N*). The corresponding routine is available in *Supporting Software*. For microscopy images, this procedure is applied for each line of image ROI (see Fig. 2, *B*–*G*, for illustration).

### Details of Other Conventional Methods

For comparison of the results of current work, we use two other conventional methods for estimating the fundamental period of a signal.

The first method, labeled as the “Fourier spectrum” method, is based on calculating the power spectrum, here defined as the square of modulo of Fourier spectrum, of a signal. The maximum point of the power spectrum is related to the fundamental spatial frequency of the signal, which, in turn, is inversely proportional to the fundamental period of the signal. For discrete signals, the power spectrum is discrete as well meaning that the fundamental period can be estimated at the pixel size resolution. To obtain fundamental period estimates at subpixel resolution, we use piecewise quadratic interpolation of the discrete power spectrum.

The second method, labeled as the “ACF” method, is based on calculating the ACF, here defined as inverse Fourier transform of the power spectrum, of a signal. The first positive maximum point of the ACF is related to the fundamental period of the signal. Similarly to the Fourier spectrum method, the fundamental period estimates are obtained at subpixel resolution by using piecewise quadratic interpolation of the discrete ACF.

In both methods, we use FFT for calculating the discrete Fourier spectra of signals. In comparison analysis of different methods we always use detrended signals as input signals to *1*) minimize boundary artifacts and *2*) ensure equal conditions for all considered methods. When applied to images, we use the mean power spectrum calculated as the average of power spectra over all image lines. The corresponding computational routines are available in Supporting Software.
